# Gene duplication as a mechanism of genomic adaptation to a changing environment

**DOI:** 10.1098/rspb.2012.1108

**Published:** 2012-10-12

**Authors:** Fyodor A. Kondrashov

**Affiliations:** Institució Catalana de Recerca i Estudis Avançats (ICREA), Centre for Genomic Regulation (CRG) and Universitat Pompeu Fabra (UPF), 88 Dr Aiguader, Barcelona 08003, Spain

**Keywords:** gene duplication, environmental adaptation, genomics

## Abstract

A subject of extensive study in evolutionary theory has been the issue of how neutral, redundant copies can be maintained in the genome for long periods of time. Concurrently, examples of adaptive gene duplications to various environmental conditions in different species have been described. At this point, it is too early to tell whether or not a substantial fraction of gene copies have initially achieved fixation by positive selection for increased dosage. Nevertheless, enough examples have accumulated in the literature that such a possibility should be considered. Here, I review the recent examples of adaptive gene duplications and make an attempt to draw generalizations on what types of genes may be particularly prone to be selected for under certain environmental conditions. The identification of copy-number variation in ecological field studies of species adapting to stressful or novel environmental conditions may improve our understanding of gene duplications as a mechanism of adaptation and its relevance to the long-term persistence of gene duplications.

## 1. Background

### (a) Motivation

Few dispute that gene duplication is the main source of functional diversity on the genotype level. The wealth of sequence data to this effect [1–3] has been preceded by theoretical considerations that it is far easier to create new functions from pre-existing ones rather than from scratch. At the heart of the interest towards gene duplications is the principle that after a duplication ‘each copy can evolve independently and diversify their effects’ [4, p. 64] leading to functional novelty [2,3,5]. As any mutation, a duplication event by itself may also have consequences on organism's fitness. However, two factors overshadowed the study of the short-term immediate fitness effects of gene duplication. First, the contrast in the difficulties in studying copy-number variation (CNV) that persist to this day [6], and the abundance of long-term evolutionary data on paralogous sequence divergence [1–3,7] shifted the attention to where the data were. Second, and perhaps crucially, the conceptual appeal of gene duplications leading to novel functions was strong enough to overshadow that of the short-term implications of duplications.

The trend of focusing on the long-term implication to the detriment of the study of short-term effects was initiated by Ohno [5] when he proposed that extra gene copies are redundant. His reasoning was that since one copy already performs the necessary function extra copies are redundant and free from selection, as they do not add anything to the organism's capacity in performing this function. As it became clearer that gene copies of various degree of divergence are present in genomes, the main question in the field became how can completely redundant gene copies be maintained in the genome long enough to evolve into a new function without being eliminated by mutation. Thus, the theoretical community made extensive use of the redundancy hypothesis with a string of papers looking at this question in some detail [8–15] with many models not formally taking account the process of fixation of a segregating gene duplication.

At the same time, data from the microbiological community were being published suggesting adaptive impacts of gene duplications under certain environmental conditions (see [16–18] for review); however, these data remain largely anecdotal and have not led to a genome-wide survey of selection on gene copy number. The diversification of the community into those that study the short-term versus the long-term effects of gene duplication may have had a linguistic component as some researchers tended to discuss ‘gene duplication’, while others studied ‘gene amplification’ with the relevant papers rarely being cross-cited. However, the fascination with the long-term consequences and the simplicity of the complete redundancy hypothesis, which essentially created the neutral theory of gene duplications, resonated in the community at the time when the neutral theory was popular and few authors took into account the adaptive point of view.

Although it is clear that some gene duplications have been fixed in the course of evolution by positive selection [16–26], whether or not it plays a significant role in the fixation of an appreciable fraction of gene duplications has yet to be comprehensively addressed at a genome-wide scale. The purpose of this review is to highlight recently described examples of adaptive short-term effects of gene duplication. Given recent reviews on whole genome duplication [27], negative impact of gene duplications [28–30] and various theories and models on gene duplications [31,32], I will avoid these issues here.

### (b) Duplication and dosage

The main issue behind whether or not positive selection for an environmental adaptation can be the driving force behind the fixation of gene duplications is whether or not gene duplications are truly redundant. Under the verbal model commonly attributed to Ohno, the variable that affects fitness is an abstract, qualitative function, such that a gene either performs a function or not [5]. By contrast, the adaptive hypothesis holds that function is a quantitative measure of gene action that can be influenced by the amount of gene product in the cell, which in turn can be influenced by the gene copy number [31,32]. However, even if the dosage of the product in the cell influences function, it is possible that a gene duplication is still quantitatively redundant if the product dosage is tightly regulated by negative feedback loops that keep the product dosage constant against gene copy number.

In the last few years, data accumulated demonstrating that while a gene duplication may not necessarily double gene dosage, it still generally leads to its increase [33–35]. It thus seems that one of the ways in which a new gene copy number may affect function is through a quantitative gene dosage effect. The complete redundancy model may still stand based on the possibility that even if dosage is increased the increase does not have an effect on fitness. The evidence against this notion is patchy and not genome-wide. However, several authors have made the claim that many CNVs are selected against in the genome owing to an increase in gene dosage, including cases of CNVs contributing to disease [28–30,36,37] and, therefore, will not be discussed here. At the very least, the data seem to show that situations when a gene duplication affects gene dosage and this, in turn, affects fitness are common. Other mechanisms that can lead to an adaptive response of a gene duplication have been described [32]; however, the dosage response is conceptually the simplest and for the purposes of this review it is assumed that the fitness effect of a gene duplication, when present, lies primarily in that the increased copy number causes an increase in protein dosage.

### (c) Adaptive gene duplications

Three genomic approaches may detect the action of selection on a specific gene duplication. Unfortunately, all three of these approaches have their limitations. First, one can study whether or not recent copies with equal function are maintained by selection by measuring the ratio of non-synonymous to synonymous evolution (d*N*/d*S*) between diverging sequences. If both are maintained by negative selection, then their initial emergence may have occurred through the action of positive selection. The logic is based on the argument that if a substitution A > G is deleterious then reverse G > A substitution is beneficial. Similarly, if in a gene copy d*N*/d*S* < 1, indicating that it is currently under negative selection and that its deletion is likely to be deleterious, then contingent on the frequency of gain-of-function mutations and lack of a new function its emergence may have been beneficial. However, when comparing very closely related gene copies with low sequence divergence, which is necessary to reduce the likelihood of this copy already performing a new function, it is not possible to measure a statistically significant departure from neutrality based on just a few substitutions, rendering the d*N*/d*S* measure relatively useless. Additionally, when comparing the d*N*/d*S* between diverged gene copies, it is possible that a measurement of d*N*/d*S* < 1 reflects selection maintaining a novel function that emerged in the process of the duplication divergence rather than selection maintaining two copies with identical function. Thus, while many studies of d*N*/d*S* between gene copies purport to reveal the action of one mechanism or other in the early evolution of gene duplications [1] such data do not necessarily provide convincing evidence for or against the action of positive selection in the fixation of such copies. Of course, it may be possible to test whether or not very recent gene copies are under selection by looking at the ratio of non-synonymous-to-synonymous polymorphisms in the same manner as d*N*/d*S* looks at the ratio of substitutions. However, in order to apply this approach, it is necessary to sequence the gene copies independently of each other, which is not possible with currently available sequencing technologies [6].

The second approach is to measure the levels of variability around the emerging gene copy and search for traces of hitchhiking effects. Only one study in *Arabidopsis thaliana* successfully applied this approach and found evidence of hitchhiking around recent gene copies [38]. However, this remains to my knowledge the only study of this type, possibly owing to the difficulties associated with assembling very recent gene duplications in whole genomes, which is generally necessary for such a study.

Finally, given enough data on fixed gene duplications and segregating CNVs, it may be possible to perform a McDonald–Kreitman test [39] to try to quantify the fraction of gene copies fixed by positive selection. This was crudely done several years ago [36], and the results remain unconvincing owing to the complications in finding a genome-wide set of accurately annotated polymorphic gene duplications to apply the test. It is likely that different genes duplicate at different rates due to difference in length [36] and the influence of short repeats that may substantially increase the rate of duplication of a DNA segment located between them [40]. This issue is coupled to the relative difficulty in identifying polymorphic versus fixed gene copies in genomes [6]. Thus, picking the right set of duplicated and polymorphic gene copies for a non-biased analysis may not be feasible at this point.

In sum, because it does not yet appear possible to systematically test the possibility of positive selection driving the fixation of gene duplications, we may study only specific cases and attempt to make generalizations based on them.

## 2. Examples of adaptive gene duplications

### (a) Transport of nutrients

Nutrient limitation has been observed in many species under different conditions and has been reviewed previously [18–21]. A clear example of a gene duplication conferring an adaptive response to nutrient limitation is that of the yeast hexose transporter. Under growth conditions with low glucose, the appearance of a new hybrid copy from two closely related paralogues, HXT6 and HXT7, increases the level of expression of the hexose transporter and, crucially, the rate of glucose transport into the cell [41]. Furthermore, the authors have shown in competition experiments that the strain with the gene duplication outcompetes the parental strain. The reason why this case is particularly exemplary is because the duplicated HXT6 and HXT7 genes are recent gene duplications themselves, with several more distantly related paralogoues in the genome [18]. It is therefore just a simple step from the experiments of Brown *et al*. [41] to the hypothesis that the paralogues HXT6 and HXT7 have appeared as a result of an adaptive duplication owing to selection favouring extra dosage of the transporters of glucose under various stressful or low-glucose environmental conditions [18,42]. Extensive experiments carried out by Gresham *et al.* [43] also show that many strains evolve amplifications either of the HXT6 or the sHXT7 gene in glucose-limited populations, although some strains adapted to starvation by different mechanisms.

### (b) Protection from heat

Adaptation to heat stress has been shown to occur through gene duplication of several stress-related genes in *Escherichia coli* [44]. Some but not all of the genes contained in the duplicated regions showed increased levels of expression and the duplication events coincided with substantial increases in fitness, although not explaining all of it. Furthermore, the duplication of evgA, a master transcriptional regulator that is a part of the signal transduction system and upregulates 37 genes, allowed *E. coli* to withstand temperatures of over 50°C [45]. The molecular mechanisms behind such robustness to heat remain unclear. Similar observations have been made in yeast and *Arabidopsis*, such that strains subjected to selection in high temperatures showed an increase propensity for chromosomal segmental duplications [46,47].

### (c) Protection from cold

A study of the genome-wide expression levels and the corresponding copy numbers provides convincing evidence for cold adaptations in Antarctic cod. In addition to the expansion of the antifreeze glycoprotein (AFGP) gene family in this species [48] a comparison of the levels of expression between the Antarctic cod and its warm water dwelling relatives revealed 177 genes with substantial overexpression in the Antarctic cod [49]. Through DNA hybridization, it was shown that 118 genes, many of which were from the same set with upregulated expression, have been duplicated in the Antarctic cod, some of them hundreds of times. Duplications were 10 times more common in Antarctic cod suggesting that upregulation through gene duplication of many different functions, including the well-characterized AFGP genes and FBP32II, an F-type lectin that is considered to be a close relative of the fish type II antifreeze proteins, played a role in cold adaptation of this species [49].

### (d) Dosage balance and restoration of fitness

Genes rarely act in isolation and it is thought that an optimal dosage of interacting proteins must be maintained for maximal fitness. This logic has been applied to explain why interacting proteins are more likely to be retained together after the whole genome duplication in yeast [50], however, it is also possible that a gene duplication may be favourable because it restores the correct dosage balance in a dosage sensitive system. Two such examples are found in the literature. In yeast, there are two pairs of closely related paralagous histone H2A and H2B proteins, coded by the HTA1–HTB1 and HTA2–HTB2 tandem genes pairs, respectively. When the dosage of the H2A and H2B drops owing to the deletion of the HTA1–HTB1 locus, one of the compensatory mechanisms restoring normal dosage and phenotype is a duplication of the paralagous HTA2–HTB2 locus [51].

Another interesting case was described by Pränting & Andersson [52]. In *Salmonella typhimurium,* a mutation in a haem-biosynthesis enzyme (hemC) increases resistance of the bacterium to protamine, however, this resistance comes at a cost of the reduction of growth. In the course of a laboratory evolution experiment, the first step of a series of events after the fixation of the resistance mutation was the amplification of the mutant hemC gene that restored some of the fitness [52]. Once the gene accumulated other compensatory point mutations that created a hemC gene that conferred resistance to protamine, but without the reduction in the rate of growth, the gene copy number was restored to normal. A similar adaptive response to the reduced fitness of a mutation leading to antibiotic resistance and a subsequent transitory compensatory increase in gene copy number of the mutated gene was found in *Salmonella enterica* [53].

### (e) Protection from salt

Adaptation to stress by high salt content may also occur through gene duplication. In one selection experiment, an increase in expression of several stress-response genes was observed, however, this increase does not appear to have anything to do with individual gene duplications as such were generally not observed in the course of the experiment [54]. However, a consistent polyploidy of the yeast strains was observed that may play a role in the adaptation [54,55]. Similarly, polyploidy has been linked to resistance to high salt concentrations in citrus [56] and sorghum [57] suggesting that polyploidy may be a general physiological adaptive response to osmotic stress [54].

### (f) Heavy metals

Duplication-induced metal resistance in different species seems to be related mainly to the export of the cations outside the cell [18,36]. Among recent publications, a study showed that *Ralstonia pickettii* adapted to high copper concentrations in copper-contaminated lake sediment tends to accumulate the copper in the outer membrane, and the genome revealed the duplication of a region with several metal resistance and transporter genes [58]. A detailed analysis of genome evolution of *Cupriavidus metallidurans*, a species particularly renowned for its tolerance of heavy metals, revealed two primary mechanisms, the increase in copy number of genes responsible for metal efflux, through HGT and duplication, and a general decrease in metal uptake [59]. A particular strain of the fungal pathogen *Cryptococcus neoformans*, var. grubii subclade VNI A5 MLST, was found to contain a tandem array of several copies of the arsenite efflux transporter that confers copy-number-correlated levels of arsenite resistance [60].

### (g) Antibiotics and drugs

Many bacteria amplify genes as an adaptive response to antibiotic treatment [36,61,62]. Similarly, it is commonly acknowledged that gene amplifications are known to occur in cancer tumours in response to various drug treatments (see Kondrashov & Kondrashov [36] for review). However, the amplification of genes in response to various drug treatments is not limited to somatic cells and microbes. In the last several years, abundant data have been collected on the amplification of genes in response to various treatments of *Leishmania* [63] and malaria [23]. The *Plasmodium falciparum* multidrug resistance gene (pfmdr1) is a target of adaptive evolution in nature in response to the widespread use of chloroquine and other anti-malarial drugs. The pfmdr1 protein is an ABC transporter involved in the transport of chloroquine outside of the cell, however, it is more directly involved in the adaptation of the malaria parasite to mefloquine, a different anti-malarial drug. Interestingly, pfmdr1 is a homologue of the human multiple drug resistance (mdr) protein that in tumours has a crucial role of expulsion of different drugs from the cell [64,65]. The pfmdr1 gene is amplified in *P. falciparum* in response to anti-malarial drug treatment, which can confer resistance to mefloquine and other anti-malarial drugs [66–68]. It is now understood that pfmdr1 gene duplication occurred independently in nature multiple times [66,69], and malaria with increased resistance to different drugs is found throughout the world from Africa [70] to Asia [71,72] and South America [73]. Finally, at least one amplification event of the pfmdr1 gene shows evidence of having occurred through the action of positive selection [74] and adaptive amplification in *P. falciparum* has been shown in at least one other gene [75], GTP-cyclohydrolase I (gch1), which is involved in the synthesis of substrates upstream of other enzymes that are commonly targeted by antifolate drugs.

Gene amplification as a mechanism of adaptation to drugs is also common in *Leishmania* [63]. The amplifications of the genes generally occur as extra-chromosomal circular DNA units [63,76], possibly due to the relatively minor role of transcription initiation in this organism [76]. This may be the reason why gene amplification and the subsequent increase in gene dosage seem to be a common basis for *Leishmania* being resistant to different drugs [77]. A genome-wide assay of *E. coli* strain in 237 toxic environments, mostly antibiotics, found 115 genes an artificial amplification of which leads to the increased fitness in the toxic environments [78]. As with the case of metal toxicity, the function that most widely corresponded to an increase in fitness under the toxic condition was that of efflux pumps and transporters closely followed by genes with regulatory functions and many of the identified genes have not been previously implicated in toxic response. Furthermore, the amplification of several genes conferred resistance in more than one toxic environment suggesting a wider role of promiscuous functions in the evolution of resistance.

### (h) Pesticides and complex organic compounds

The application of pesticides and herbicides promotes the adaptive duplication of enzymes that digest the chemical ([79] and see [25] for review). A well-studied example is that of the mosquito *Culex pipiens* that was subject to pesticide treatment on the southern coast of France. Adaptation to pesticide treatment evolved by the duplication of two non-specific esterases (Est-2 and Est-3), and Ace.1, a locus encoding acetylcholinesterase that is the main target of the applied organo-phosphate insecticides [36,80]. However, the duplication of Ace.1 comes at a substantial fitness cost [80], which has led to intricate evolutionary dynamics in the evolution of this locus with subsequent duplications being maintained in heterozygous states across the population owing to a low fitness of the duplications in homozygous state [81]. Furthermore, similar to the strong selection pressure of the anti-malarial drugs leading to independent multiple duplications of the pfmdr1 gene, the Ace.1 resistance duplication also appeared multiple times independently around the world [82,83]. The combination of independent origin and the persistence of the duplication in heterozygous form despite the low fitness of homozygous states indicate positive selection for the original emergence of the heterozygous duplication.

### (i) Adaptation to domestication

Different cow breeds have been analysed for differences in CNVs with different functional families featuring prominently in the differences. First, CNVs of immune-related genes coding for UL16-binding proteins have been described in different breeds of cattle [84,85] and fixed copies of these genes have been shown to be duplicated in the recent ancestor of the *Bos* lineage [86]. Furthermore, recently duplicated genes have been associated with recent domestication-related phenotypes [86] such as milk proteins and proteins related to ruminant digestion. A recent genome-wide study of three cattle breeds identified approximately 400 genes located in CNVs, many of which were related to different environmental interaction and domestication related functions, including parasite and pathogen resistance, lipid transport and metabolism [85]. In a few cases, the types of gene function could be linked to breed-specific phenotypes, suggesting that several aspects of cattle health and productivity have been selected through CNV in the course of cattle domestication.

### (j) Adaptive duplications in humans

In our own species, several examples have been described of a polymorphic duplication conferring either a distinct phenotype or a fitness advantage. Thus, individuals with more copies of the CCL3L1 gene, a ligand for the HIV-suppressive CC chemokine receptor, have lower susceptibility to HIV infection and slower progression to AIDS after infection [87]. A widely reviewed example is that of the salivary amylase gene (AMY1) that plays a role in the initiation of starch digestion. Individuals from populations with higher starch content in their traditional diets have a higher copy number of this protein providing an apparent fitness benefit [88]. The high prevalence of CNV in the human genome coupled with data that many of them may be adaptive [37,89–91], suggest that more examples of adaptive gene duplication in the human population are yet to be described.

### (k) Generalizations

Adaptive duplications in different species continue to be described in the literature (see table 1 for additional examples) implying that gene duplication as a form of adaptation to various environmental conditions is not a rare mechanism, although by far not an exclusive one. Perhaps several generalizations can be made regarding when an adaptive duplication may be expected to play a role in various adaptations in nature. First, in instances when the protein products of the genes physically interact with molecules associated with a variable environment such as toxins or nutrients. Second, when the proteins coded by the duplicated genes function in the same pathway as those that physically interact with such molecules. Finally, adaptive duplication seems to involve those genes which product needs to be produced rapidly or constantly at a high level, such as antifreeze or storage proteins.

**Table 1.** Recent examples of adaptive gene duplication not discussed in the main text.

gene	function	species	environmental condition	evidence	references
Lac operon	lactose metabolism	*Escherichia coli*	starvation	laboratory experiments	[92–94]
SUL1	high affinity sulphur transporter	*Saccharomyces cerevisiae*	low-sulphur environment	laboratory experiments	[43]
prolamine-coding genes	storage proteins	Poaceae	nutrient storage in seeds	indirect inference from large number of copies	[95]
AFP	antifreeze protein	*Anarhichas lupus*	low temperature	higher number of copies and higher expression in a cold-tolerant species	[96]
IRI-like gene family	ice re-crystallisation inhibition	Pooideae	low temperature	gene family expansion in cold-tolerant lineage	[97]
COR15	cold tolerance-related protein	Brassicaceae	low temperature	several independent duplications	[98]
ribosomal protein coding genes	protein translation	*Salmonella typhimurium*	horizontal gene transfer	laboratory experiment	[99]
EPSPS	5-enolpyruvylshikimate-3-phosphate synthase	*Amaranthus palmeri, Medicago sativa, Glycine max, Nicotiana tabacum,* other species	glyphosate	field studies, experimental evidence	[26,100–102]
Gpdh	glycerol-3-phosphate dehydrogenase	*Drosophila melanogaster*	ethanol	laboratory experiments with artificial amplification	[103]

The main logic for hypothesizing that fixed gene duplications played an adaptive role in dosage response to stressful environment has been the functions of gene duplications with characterized adaptive role and the functions of fixed gene copies that are observed in the genome [16–18,21,22,36,104–116]. However, it may be instructive to reverse the logic and predict the types of genes that may confer an adaptation in some environmental conditions based on the functional repertoire of the gene duplications observed in the genome.

One of the main duplicated gene families are the olfactory receptor proteins [18,117–119] so perhaps their duplication may lead to an increase in sensitivity to a particular odour may be adaptive under certain conditions. The match in the sensitivity range of duplicated opsin genes and coloration [120,121] suggests that the duplication of an opsin gene may cause an adaptive increase in sensitivity to light of a certain wavelength. Duplications of genes involved in pathogen resistance [105,108–111,122,123] and pathogenicity [110,124] suggest that gene duplications may have a role in rapid coevolution between host and pathogen or symbiont. Recent gene duplications in drought-resistant plants [125] suggest that genes involved in osmotic stress response are also good candidates. Duplication of the globin genes [107,126] may be adaptive in low-oxygen environments, such as high altitude, by optimizing oxygen transport. Common duplications of different defence toxins and venoms [116,127–132] suggest that toxin dosage and their effectiveness may be regulated through gene duplication. Similarly, the duplication of proteins involved in the degradation of toxins, for example enzymes that neutralize pyrrolizidine alkaloids [133], may confer a fitness advantage to a herbivore feeding on a plant with high concentration of specific toxins. The duplication of developmentally related genes [134] may confer an adaptive advantage in the anatomical modification of the body plan for a new environment. The production of carotenoids [135] or light emission by luciferase [136] may be dosage-mediated by gene duplication leading to extra pigment or higher intensity of light. The duplication of proteases expressed in the female reproductive tract of *Drosophila mojavensis* [137] and sperm-related proteins in *Caenorhabditis elegans* [18] suggests that dosage increase through gene duplication may be adaptive as a strategy in sexual conflict.

## 3. Discussion

The development of the theory of gene duplications reflects that of the neutral theory of molecular evolution. The strong claim of neutrality of gene duplications made by Ohno [5] reflected a point of view that all gene duplications are neutral. Most theoretical models that followed maintained that the redundancy of the gene duplication leads to its neutrality. In the last few years, it became evident that many gene duplications are deleterious from the moment of their origin [28–30,138]. However, this can be easily accommodated in the redundancy-based theories on long-term evolution of gene duplication in the form of a weak claim of neutrality: those gene duplications that are fixed are neutral. Because no quantitative genome-wide estimates of the fraction of gene duplications that are fixed by positive selection have been made, population genetic theories explaining the evolution of gene duplication have not incorporated selection on gene copy number in the models [31,32].

At the present moment, the literature reflects a point of view that gene duplications are more neutral than amino acid substitutions. A large fraction of amino acid substitutions is thought to be fixed by positive selection [139], yet most authors continue to explain the origin and maintenance of gene duplications by referring to theories of their maintenance that assume complete redundancy and a neutral path to fixation. However, presumably a gene duplication is mutation of stronger impact than a point mutation and, therefore, it should on average have a more profound impact on fitness than a substitution [19,20]. This somewhat paradoxical situation is reflected in the literature on CNVs where authors avoid reference to the existing theories on gene duplication [28–30,36,37].

Owing to the fascination with the idea of complete genetic redundancy, classical population genetic and evolutionary models of gene duplications generally failed to be useful in the description of CNV in natural population (but see [140–142]). Perhaps the same fate of irrelevance awaits the theoretical field once ecological genomics starts addressing the question of adaptation to stressful environmental conditions on a genome-wide scale in multiple non-model organisms. To model such adaptation, the necessary theoretical models may be more complicated than those simply incorporating a quantitative measure of genetic redundancy as they may also have to be time-dependent; in several cases, it appears that a gene duplication that is adaptive under a stressful condition comes at a fitness cost in a benign environment [36,52,80,143,144].

The cost and utility of sequencing technology makes it possible to obtain a genome sequence of reasonable quality for all but the most complicated genomes. The application of such technology to natural populations is bound to reveal new mechanisms of ecological adaptation. Given the abundance of examples of adaptive gene duplications that emerge in the course of adaptation to stressful environments, researchers studying the adaptation of species to novel or stressful environments would be well advised to consider taking a closer look at recent gene duplications and copy-number polymorphisms. Meanwhile, the relative role of positive selection versus drift in fixation of gene duplications remains an open question.
